# Dynamic changes of timing precision in timed actions during a behavioural task in guinea pigs

**DOI:** 10.1038/s41598-020-76953-y

**Published:** 2020-11-18

**Authors:** Masataka Nishimura, Chi Wang, Reika Shu, Wen-Jie Song

**Affiliations:** 1 Department of Sensory and Cognitive Physiology, Faculty of Life Sciences, Kumamoto University, Kumamoto, Japan; 2 Program for Leading Graduate Schools HIGO Program, Kumamoto University, Kumamoto, Japan

**Keywords:** Physiology, Behavioural methods, Decision, Attention

## Abstract

Temporal precision is a determinant of performance in various motor activities. Although the accuracy and precision of timing in activities have been previously measured and quantified, temporal dynamics with flexible precision have not been considered. Here, we examined the temporal dynamics in timed motor activities (timed actions) using a guinea pig model in a behavioural task requiring an animal to control action timing to obtain a water reward. In well-trained animals, momentary variations in timing precision were extracted from the temporal distribution of the timed actions measured over daily 12-h sessions. The resampling of the observed time of action in each session demonstrated significant changes of timing precision within a session. Periods with higher timing precision appeared indiscriminately during the same session, and such periods lasted ~ 20 min on average. We conclude that the timing precision in trained actions is flexible and changes dynamically in guinea pigs. By elucidating the brain mechanisms involved in flexibility and dynamics with an animal model, future studies should establish more effective methods to actively enhance timing precision in our motor activities, such as sports.

## Introduction

Higher temporal precision is crucial for the performance of motor activities, which require precise timing in each action, such as handcrafting, machine operations, and sports. One example in sports is timing of the take-off movement for a ski-jump, which is an important determinant of jump length^1^. To study the brain mechanism involved in timing processing for such actions, the accuracy and precision of timing in timed actions have been measured and quantified in animals and humans (reviewed in^2^). Based on the behavioural results, computational models to decide on the time to perform an action, such as the scalar timing model, have been proposed in previous studies^3–6^. In these behavioural and computational studies, however, timing precision has implicitly been considered to be inflexible and moment-by-moment changes in timing precision (or dynamics of timing precision) have not been considered in the analyses. Importantly, both the accuracy and precision of the moment at which the action is taken has a critical impact on achieving optimal timing for the desired action, as in the example of ski-jumps.

Extraction of the steps leading to timing precision, in principle, requires measurement of the distribution of the action time. Therefore, two factors are required for extrapolating the dynamics of precision in timing: (1) a high density of temporal samples of action time, to extract momentary timing precision over a sufficiently large distribution of action times and (2) a large number of action times over a prolonged period, to trace temporal changes in timing precision. To satisfy these requirements, we designed a behavioural task for guinea pigs whereby they perform timed actions based on operant conditioning, with each activity session lasting 12 h and implemented daily. Unlike Pavlovian conditioning (or classical conditioning), operant conditioning can be applied to increase the density and number of samples of action times obtained, because animals voluntarily continue to perform the task to obtain rewards unless motivation is lost. In the behavioural task we designed, in order to suppress reflexive or impulsive actions^7,8^, one of two different cues was presented to the animal in a self-initiated task activity and the animal had to attentively control the timing of his action based on his capacity for cue discrimination in order to obtain the reward. Our results demonstrated the flexibility and dynamics of timing precision in timed actions during the task in well-trained guinea pigs. The brain networks and mechanisms involved in the flexibility and dynamics can be explored in future studies using a guinea pig model.

## Results

### Self-timed action learning through a behavioural task

**Figure 1:**
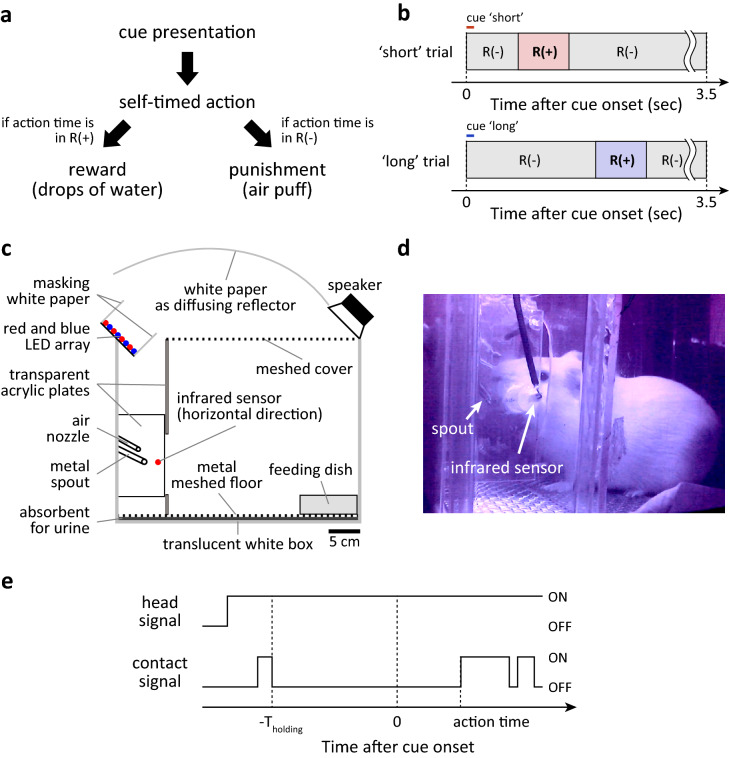
The behavioural task used in this study. (**a**) Task flows to reward or punishment in each task trial. (**b**) Examples of the R(+) time window in ‘short’ trials and in ‘long’ trials in the timeline of a single trial. (**c**) Schematic illustration of the operant conditioning chamber for the behavioural task. All items are illustrated in a proper scale with the scale bar. (**d**) A picture of guinea pig waiting for the next cue near the spout, which was taken with an infrared sensitive camera under the infrared illumination attached to the top of the chamber. (**e**) An example of head and contact signals around the cue onset. If the head signal is ON, the infrared sensor is detecting an object, e.g. head, masking the horizontally aligned infrared light. If contact signal is ON, there is a physical contact between the animal and spout. T_holding_ is holding time to trigger a cue presentation.

We designed a behavioural task using operant conditioning to train guinea pigs to perform a timed action without providing them with a reference time before the action or designing the task as a self-timed activity^7,8^. The principle of the behavioural task was that the animal was to receive a reward or punishment depending on time of action required after cue presentation, or action time, for each trial of task (Fig. 1a). If the action time was within a pre-established time window for giving a reward, called the R(+) time window, drops of water through a spout would be delivered as a reward. If the action time was outside of the R(+) time window, i.e., in the R(–) time window, an air puff would be delivered as punishment (Fig. 1a). The time of first contact between the animal and spout after the cue onset was defined as the action time in the trial. To suppress reflexive or impulsive actions^7,8^, one of two randomly chosen cues, called the ‘short’ cue and the ‘long’ cue (Fig. 1b), was presented in each trial of task. Because trials with different cues principally had one of two different R(+) time windows (Fig. 1b), the chances of obtaining a reward in a trial of task would be higher if the animal attentively controlled timing of action based on cue discrimination. The tasks were conducted in a newly designed operant conditioning chamber (Fig. 1c) inspired by the Skinner box^9^. To suppress attention-less or random actions, the guinea pig was required to keep its head steady near the waterspout for an average of 2 s, without any licking, to initiate a trial (Fig. 1d,e).

**Figure 2:**
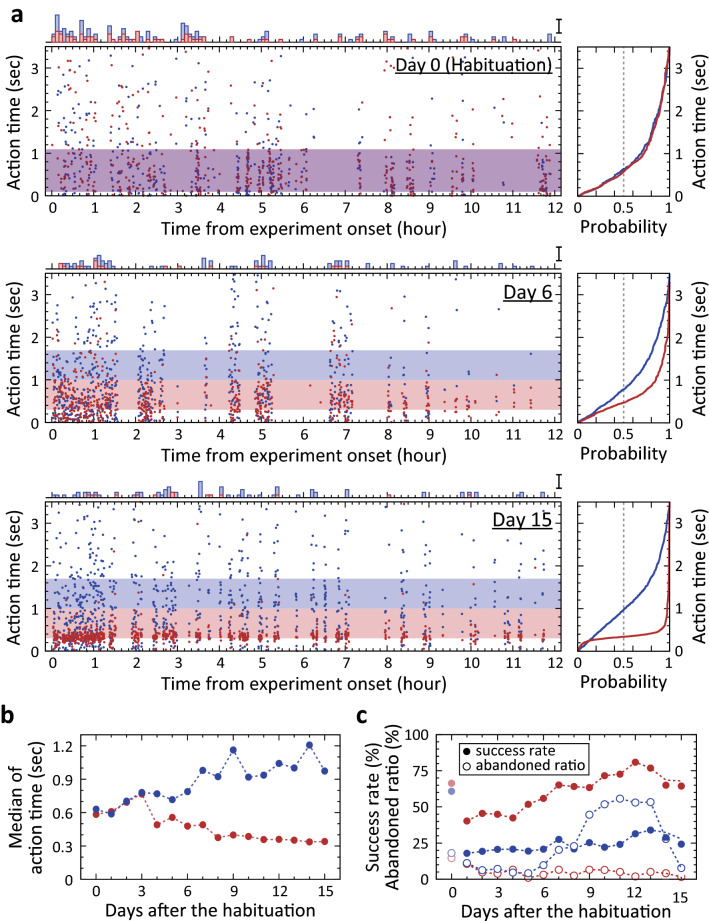
Learning process of the task in a representative guinea pig. (**a**) Action time for each trial of the task and its cumulative distribution. Left panels: Raster plots of action time on Days 0, 6, and 15. Red and blue dots indicate the time in ‘short’ and ‘long’ trials, respectively. This colour assignment for these cues is applied to all panels in this figure. The horizontal purplish band in Day 0 shows the overlapped R(+) time windows for ‘short’ cue and ‘long’ cue, 100–1100 ms. The horizontal reddish and bluish bands in Day 6 and Day 15 show R(+) time windows for ‘short’ cue and ‘long’ cue, respectively. These were 300–1000 ms and 1000–1700 ms, respectively. Histograms on each raster plot show the number of abandoned trials for each cue in the day. Bin width is 5 min. Scale bar is 5 abandoned trials. Right panels: Cumulative distribution of action time for each cue. The intersection of vertical dashed line and the probability curve is the median shown in (**b**). (**b**) Gradual separation of the two medians of action time for these cues in the guinea pig. (**c**) Change of success rate and abandoned ratio over days of experiment in the guinea pig. Filled and open circles indicate the success rate and abandoned ratio, respectively. The rate and ratio in Day 0 (habituation) are shown in faint colours.

One day was given to the animal for habituation to the task, in which cue discrimination was not required. This was essential to ensure that naïve animals could learn to perform the task. Guinea pigs learned to lick the spout in response to a presented cue over in several hours, as evidenced by the decrease in the number of “abandoned” trials, in which no contact between animal and spout was detected by the deadline of a trial of task (3.5 s after cue onset), during the day of habituation (Fig. 2a; Day 0). For further statistical analysis of the learning within 1 day of habituation, we analysed the abandoned ratio, which is the number of abandoned trials divided by the total number of trials, and success rate, which is the number of rewarded trials divided by the number of performed trials, in moving 2-h time-windows (Supplementary Fig. S1a,b). The abandoned ratio and success ratio in this analysis time-window at the early habituation period (1–3 h in Supplementary Fig. S1a,b) was significantly inversely correlated in the discriminated animals described below (n = 12; Pearson's correlation coefficient: r = – 0.450 ± 0.165, p < 0.05, t-test and sign test; Supplementary Fig. S1c), indicating that these guinea pigs decreased the rational number of non-specific licking behaviours during the early habituation period. After the habituation period, the guinea pigs gradually learned to control their action time based on the discrimination of cues (Fig. 2a, right panels, from Day 0 to Day 15). Distribution of the action time in ‘short’ trials and in ‘long’ trials were significantly different in 12 of the 16 tested animals (Kolmogorov–Smirnov test, p < 0.05), and a significant difference was repeatedly observed in following daily sessions, once the p-value fell below 0.01 in the Kolmogorov–Smirnov test. Approximately 4.2 ± 2.0 days after the habituation session (n = 12, error in SD) had passed before the p was < 0.01 in the Kolmogorov–Smirnov test, and a stable difference in the medians was observed (Fig. 2b). The learning curve in the medians appeared to reach a plateau within 10 days (Fig. 2b), similarly to that in the interval discrimination tasks in human subjects^10–12^. A progressive change in the distribution of action times in the ‘long’ trials appeared less obvious than that in ‘short’ trials in the tested animal (Fig. 2a, right panels for a representative example). To quantitatively describe the learning process, we defined the success rate as the number of rewarded trials divided by the number of total trials, except abandoned trials, and the abandoned ratio, as the number of abandoned trials divided by the total number of all trials. These two indices were calculated for each cue in each daily session. The success rate in ‘long’ trials gradually increased from ~ 20 to ~ 30%, while the success rate in ‘short’ trials increased from ~ 40 to ~ 70% over 2 weeks of training (Fig. 2c, filled circles). The abandoned ratio in ‘long’ trials became consistently higher than that in ‘short’ trials after 11 consecutive days, as the training progressed (Fig. 2c, from Day 5 to Day 15; p < 0.01, sign test). This difference in the abandoned ratio with the concomitant increase in the success rate was further evidence that the animal had learned to discriminate cues for its actions. This learning process in naïve animals did not change substantially, irrespective of the use of auditory or visual cues. The time after the habituation session until significant differences in action time distribution were observed (p < 0.01, Kolmogorov–Smirnov test) was 4.4 ± 0.78 days with auditory cues (n = 8) and it was 3.5 ± 0.87 days with visual cues (n = 4), with the difference not being significant (p > 0.05, Mann–Whitney U test). Thus, data obtained in animals performing the task using auditory or visual cues were analysed as results obtained from the same task.

**Figure 3:**
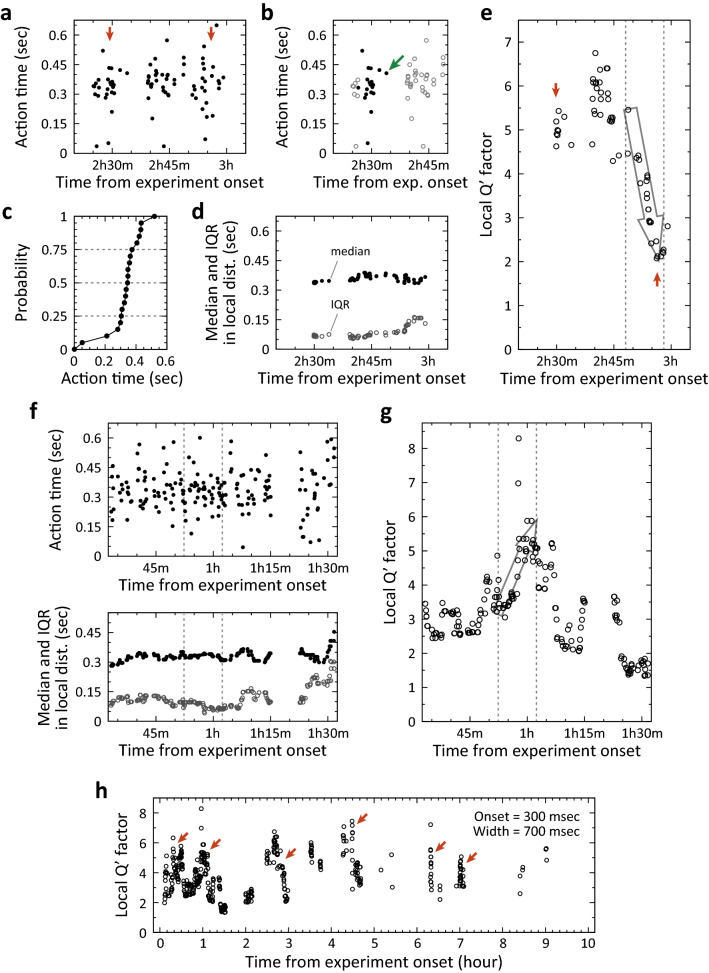
Varied deviation of action time and extracted rapid changes in local Qʹ factor. (**a**) Subset of action time in ‘short’ trials from 2 h 20 m to 3 h 5 m after the onset of experiment on Day 15 shown in Fig. 2a. Arrows on the left and on the right point to samples of action time with smaller deviation and samples with larger deviation, respectively. (**b**) A collection of 20 consecutive samples of action times from the target trial that is indicated by the arrow. Filled circles are samples obtained during data collection. Open circles are other samples, not belonging to the data collection. (**c**) Cumulative distribution of action time in the data collection shown in (**b**). (**d**) Median and interquartile range (IQR) measured in local distributions of action time as exemplified in (**c**). Black filled circles and grey open circles indicate median and interquartile range (IQR), respectively. (**e**) Rapid decrease of local Qʹ factor in the period shown in (**a**). Arrows on the left and on the right point to the local Qʹ factors extracted from the samples pointed by arrows in (**a**). The interval between vertical dashed lines is 10 min. The open arrow represents approximate direction of local Qʹ factor change in the period. (**f** )The same analysis in a different period in the same daily session. The interval between vertical dashed lines is 10 min, also in (**g**). (**g**) Rapid increase of local Qʹ factor in the period shown in (**f**). Open arrow represents approximate direction of local Qʹ factor change in the period. (**h**) Temporal change of local Qʹ factor during the task on Day 15 shown in Fig. 2a. Arrows point to rapid changes of local Qʹ factor. The onset and width of R( +) time window in ‘short’ trials were 300 and 700 ms, respectively.

We examined the temporal dynamics of timing precision in well-trained animals. Four of the 12 animals were trained for more than 2 weeks and were defined “well-trained animals”. The onset of the R(+) time window for ‘short’ cue was 200 ms or 300 ms after the cue onset, and the onset of the R(+) time window for ‘long’ cue was 1000 ms after the cue onset, in all well-trained animals. The width of the R(+) time window was the same for both cues, ranging from 200 to 700 ms. The onset and width of the R(+) time windows were fixed for each well-trained animal. We aggregated data from well-trained animals trained with different onsets and R(+) time window widths in the following analyses because these differences had no obvious impact on dynamics as exemplified by data of local Qʹ factor (Supplementary Fig. S2, compare to Fig. 3h), which is described in the next section.

### Temporally varied deviations in action time in well-trained animals

Trial-by-trial deviations of the action time appeared to vary over time within a daily session in well-trained animals. Figure 3a shows the data obtained over a period of 45 min for the example shown in Fig. 2a (Day 15). The arrows on the left and right in Fig. 3a indicate samples of action time in ‘short’ trials with smaller trial-by-trial deviations and samples with larger trial-by-trial deviations, respectively. To quantify this observation, we measured the median and interquartile range (IQR) of the local distribution of 20 consecutive samples of action time (Fig. 3b,c for an example; see “Methods” for sample collection rule). In the period shown in Fig. 3a, the change in the IQR was greater than 200% with less than a 10% change in the median value (Fig. 3d). To mathematically describe timing precision, we introduced a time version Q factor, called the Qʹ factor (Qʹ = T/ΔT; T and ΔT are the median and IQR in Fig. 3d,f). Although the population distribution of action times must be identified to calculate the Qʹ factor, the population distribution cannot be identified based on a local distribution due to the limited number of samples. Thus, we calculated a “local Qʹ factor” as the estimated Qʹ factor for each moment, by dividing the median (as estimated T in the Qʹ equation) by the IQR for each local distribution of action times (as the estimated ΔT in the Qʹ equation). A rapid change in the local Qʹ factor of ~ 10 min (Fig. 3e,g) was frequently observed in well-trained animals and sporadically observed during the daily sessions, as exemplified in Fig. 3h.

### Significant temporal changes in timing precision

**Figure 4:**
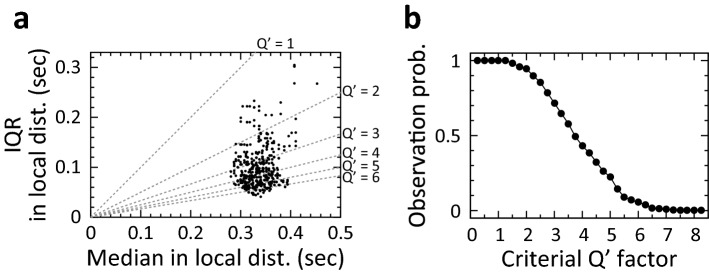
Calculation of the probability of observing a local distribution in which local Qʹ factor exceeds a local Qʹ factor threshold in a daily session. (**a**) Scatter plot of median and interquartile range (IQR) measured in each local distribution of action times in the same ‘short’ trials on Day 15 shown in Fig. 2a. The dashed line indicates median and IQR to be the local Qʹ factor. (**b**) Probabilities to observe a local distribution of which local Qʹ factor exceeds the local Qʹ factor threshold in the same daily session are shown in (**a**). The probability was calculated at 0.25 steps of local Qʹ factor threshold.

**Figure 5:**
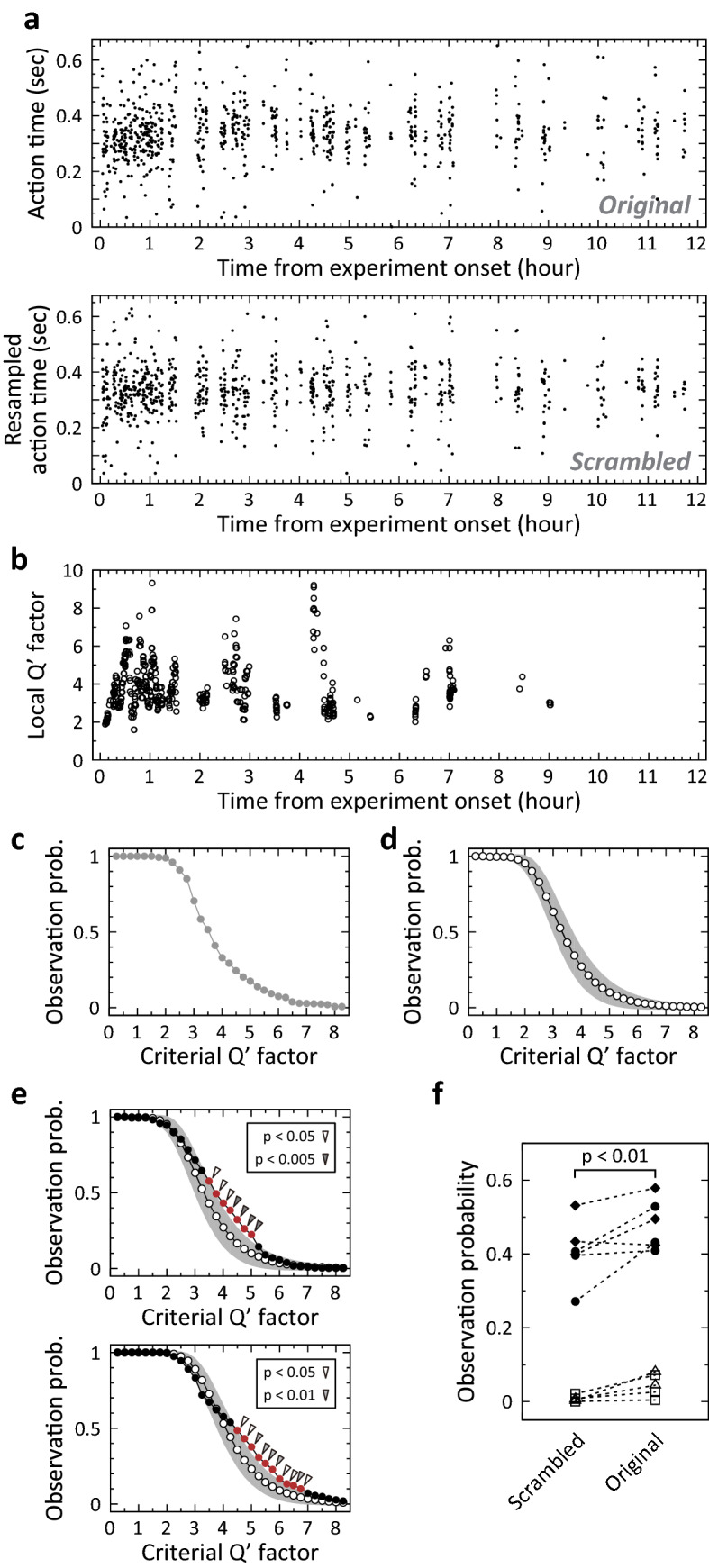
Statistical analysis of the observation probability by resampling the action time. (**a**) One resampling trial was obtained by data scrambling. The upper panel shows the observed action time in the original data. The lower panel is the resampled action time in scrambled data. (**b**) Extracted local Qʹ factors from the scrambled data shown in (**a**). (**c**) Probabilities to observe a local distribution of which local Qʹ factor exceeds the local Qʹ factor threshold in the scrambled data shown in (**a**). (**d**) Calculated possible range of observation probabilities under the assumption of no temporal change of timing precision. Open circle and greyish range indicate the mean of probability and the 95% confidence interval, respectively. The possible range was calculated from 10,000 sets of resampling and observation probability analysis. (**e**) Upper panel: A statistical analysis of the observation probability in a daily session obtained from a well-trained animal, compared to the possible range. The filled circle indicates the observation probability in original data that is shown in Fig. 4b. The possible range is shown in the same manner as (**d**). Significantly higher probability is marked by the arrowhead. Open arrowhead indicates p < 0.05. Double arrowhead indicates p < 0.005. Lower panel: The same examination in a different well-trained animal. The grey arrowhead indicates p < 0.01. (**f**) Comparison of probabilities to observe a local distribution of which local Qʹ factor exceeds 4 in scrambled data (left) and in original data (right), pooled from the four well-trained animals. One pair of probabilities linked with the dashed line was extracted from a daily session in an animal. Different symbols indicate different animals. The animals shown in (**e**) are represented by circle and diamond. The p-value was determined by the Wilcoxon signed-rank test (n = 11).

The observed temporal change in the local Qʹ factor may be a result of the potential variability of the local Qʹ factor, which was calculated using a small number of samples, without a change in timing precision (Supplementary Fig. S3). To examine the statistical significance of the observed change, we first measured the number of local distributions in which the local Qʹ factor exceeded the threshold, named the local Qʹ factor threshold, during a daily session (Fig. 4a). Next, we calculated the probability of observing a local distribution in which the local Qʹ factor exceeded the local Qʹ factor threshold, by dividing the number of local distributions by the total number of all local distributions during the daily session, at 0.25 steps of the threshold (Fig. 4b). To estimate the probability of observing such local distributions when there was no temporal change in timing precision, we resampled the action time in the daily session by scrambling all samples of action time in the session (see “Data scrambling” in “Methods”) (Fig. 5a). For one set of scrambled data, the local Qʹ factors (Fig. 5b) and the observation probabilities (Fig. 5c) were calculated. This process was repeated 10,000 times, and the mean observation probability and its 95% confidence interval were calculated for each local Qʹ factor threshold (Fig. 5d). During the representative daily session shown in Fig. 5a, the observation probability was significantly higher than that in the scrambled data when the local Qʹ factor threshold ranged from 3.5 to 5 (Fig. 5e, upper panel; p < 0.05, open arrowheads; p < 0.005, double arrowhead). The lower panel of Fig. 5e shows another example of significantly higher observation probability in another well-trained animal. Although a significantly higher observation probability was frequently observed in the ‘short’ trials, it was rarely observed in the ‘long’ trials. Thus, we focused on the action time in the ‘short’ trials for the following analyses.

Although all of the four well-trained animals showed significant changes in timing precision with the analysis described above, statistical significance over animals and sessions remained unclear. To examine the significance, the same observation probability analyses were conducted during the last three daily sessions in these well-trained animals. To compare probabilities using the same criteria across different animals, a single local Qʹ factor threshold, 4, was used to calculate the observation probabilities in the original data and in the scrambled data (Fig. 5f, n = 11 sessions, 3 sessions from three animals and 2 sessions from one animal). One session relative to one animal was excluded due to the lack of local Qʹ factor exceeded the threshold. The observation probability in the original data was significantly higher than the mean values in 10,000 sets of resampled data (Wilcoxon signed-rank test, p < 0.01). These results indicated that significant temporal changes in timing precision occurred in well-trained animals and timing precision in the trained actions were flexible.

### Occurrence and duration of higher timing precision periods in daily sessions

**Figure 6:**
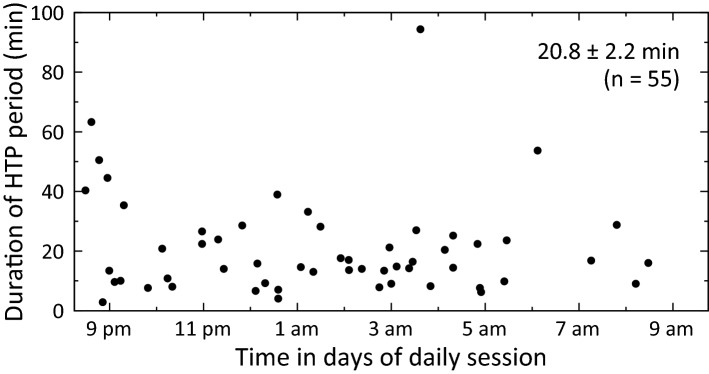
Analysis of appearance times and duration of higher timing precision periods in the four well-trained animals. Onset and duration of higher timing precision (HTP) period, measured in the 11 sessions shown in Fig. 5f from the four animals. One dot marks the onset of HTP period in horizontal axis and duration of HTP period in vertical axis. Fifty-five HTP periods in total were extracted from these animals. Duration of HTP period was 20.8 ± 2.2 min (error indicated as standard error of mean [SEM]).

Because each daily session lasted 12 h, a significant temporal change in timing precision in a session might not have been attributable to fast dynamics but to a progressive change in timing precision over the 12-h period or to a slow modification of internal physiological properties involved in time processing^13^. To examine this possibility, we extracted higher timing precision (HTP) periods from the ‘short’ trials from the same population data for the 11 sessions described above. An HTP period was defined as the period in which any local Qʹ factor did not fall < 4 and the time interval between two neighbouring ‘short’ trials was less than 15 min. All consecutive trials satisfying the criteria were merged into an HTP period to measure the onset and duration of the HTP period. HTP periods appeared randomly during the daily task session (Fig. 6). The duration of HTP period was 20.8 ± 2.2 min (n = 55, error in SEM) and there was no obvious change of duration over the 12-h period, except at the onset of some sessions (Fig. 6).

## Discussion

We demonstrated flexibility and dynamics of timing precision in trained actions of guinea pigs by analysing the temporal distribution of action times. Although the number of well-trained animals for the statistical examinations was limited to four, this number appeared to have no crucial influence on the statistical conclusions because statistical significance of the dynamics was observed using both individual data from each animal (Fig. 5e) and pooled data from all animals (Fig. 5f). One reason for the successful extraction of dynamics of timing precision is the conditioning protocol. Traditionally, Pavlovian conditioning has been applied to measure accuracy and precision of action timing in animals^2^. However, it has the disadvantage of increasing the density and number of samples required to measure the distribution of action time, because probe trials without reinforcement, which appear at low probabilities in the task session, are required for measurement. In comparison to Pavlovian conditioning, it is possible to simply increase the density and number of samples by voluntary performance of the task, unless the animal loses motivation, by introducing operant conditioning. Although recent studies in monkeys have applied operant conditioning to measure the distribution of action time in self-timed actions^7,8,14^, these studies have not focused on the dynamics of timing precision.

In the behavioural task used in this study, the dynamic change of timing precision was rarely observed in ‘long’ trials, although it was frequently observed in ‘short’ ones. This result may be explained by the difference in success rates in ‘short’ and ‘long’ trials. By considering the scalar expectancy theory^3^, the error range of action time would be larger with longer action times. If the widths of the R( +) time window were equal in ‘short’ and ‘long’ trials as in the present study, the success rate in ‘long’ trials would be smaller than that in ‘short’ trials as exemplified in Fig. 2c. Indeed, the success rate in ‘short’ trials in the four well-trained animals was approximately three times higher than that in ‘long’ trials. The higher success rate, or higher probability to obtain a reward, in ‘short’ trials might have provided higher motivation to temporarily enhance timing precision.

A higher timing precision period lasted for ~ 20 min on average, and such periods appeared at sporadic time points during a session (Fig. 6). The fluctuation of timing precision was much more rapid than circadian fluctuation of time perception (minutes vs. hours)^13^, indicating that guinea pigs temporarily enhanced timing precision during the session. The temporary enhancement of timing precision may be attributed to short-term enhancement of concentration level on the task for behavioural output, together with enhanced attention level to the cue for sensory input. Longer duration of higher timing precision period at the onset of some sessions, 40 to 60 min (Fig. 6), may have been observed due to the dehydration at the onset that induced higher motivation for the enhancement of attention and concentration levels. Attention-related neuronal activities and networks have been identified in previous studies^15–21^. It would be interesting to examine whether and how attention-related cortical networks are involved in the dynamics of timing precision in future studies. By elucidating the brain mechanisms involved in the flexibility and dynamics using guinea pig as a model, scientifically-reasonable objective methods for humans beyond experience-dependent subjective approaches to actively enhance timing precision in motor activities, such as handcrafting, machine operation, and sports, may be established in the future.

**Figure 7:**
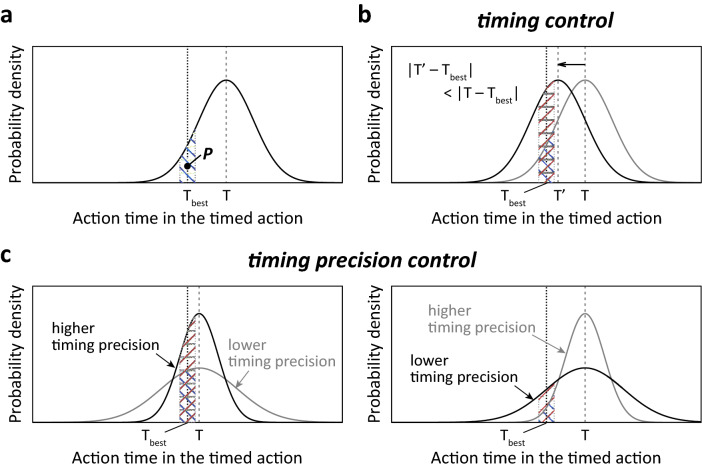
Control of timing and timing precision for “better timed action”. (**a**) An example of probability density function of action time in the timed action to achieve the best timing (T_best_) to perform the action. T is timing achieved in the timed action, or action timing. P is the probability to observe the action time around T_best_. (**b**) Timing control to increase P. T′ is the action timing after timing control. (**c**) Timing precision control to increase P.

How do the flexibility and dynamics affect the performance of timed actions? In timed actions of animals and humans, there is an optimal time to act, defined as the best timing (T_best_). Action timing (T) will not always be T_best_ if the limitation of timing accuracy is considered (Fig. 7a). The probability to observe action time around the T_best_ (P) can be calculated by the integral of the probability density function of action time around the T_best_ (Fig. 7a). If we define timed action with higher P as “better timed action”, there are two possible targets of control for “better timed action” as the issue of accuracy and precision in general timing control system. One target of control is action timing (Fig. 7b). If action timing after timing control (T′) is closer to T_best_ with no change in timing precision, P will be increased by the control of action timing. Another target of control is timing precision (Fig. 7c). When T is close to T_best_, increasing timing precision will increase P (Fig. 7c, left panel); conversely, decreasing timing precision will increase P, when T is distant from T_best_ (Fig. 7c, right panel). Because the speed of the internal clock in the brain is proposed to be modulated by several physiological factors such as arousal level and neuromodulators^7,8,14,22–29^, T may become more distant from T_best_ in some situations even after a long history of training. In this case, animals can decrease timing precision to increase P and it would increase chance to know T_best_ for the timing control from the result of the timed actions (Fig. 7c, right panel). After the timing control with decreased timing precision, animals can choose whether to increase timing precision to further increase of P (Fig. 7c, left panel). According to this scenario, timing precision should be decreased or increased for “better timed action”, depending on the difference of T from T_best_. Taken together, the flexibility and dynamics of timing precision can contribute to improving the performance of a timed action in variable clock conditions in the brain or with an impaired timing control.

## Methods

All animal experiments were performed according to the Regulations for Animal Experiments at the Kumamoto University. The protocol was approved by the Committee of Animal Experiments of Kumamoto University. We used Hartley guinea pigs for the experiments (Kwl:Hartley or Slc:Hartley; 16 males, > 6-weeks-old).

### Operant conditioning chamber

In principle, the chamber was designed for larger rodents such as guinea pigs and rats to precisely measure the time of action to lick the spout. Physical contact between the animal and spout for the action was detected by a change in electrical resistance from the spout to the floor with a < 0.1 ms detection latency. The inner diameter of the spout was ~ 0.8 mm. Animals had ad libitum access to food pellets from the feeding dish in the chamber (Fig. 1c). Water from a reservoir tank, in which the water level was controlled to achieve a flow speed 12.0 ± 0.3 mL/min, was gated near the spout with a medical grade solenoid valve for liquid (UMB1-T1-DC12V, CKD, Aichi, Japan). The insertion of the animal’s head into the hole leading to the spout was detected with an infrared sensor (Fig. 1c,d). If the animal touched the spout except for a rewarded case (Fig. 1a), an air puff (0.5 MPa, 10 ms) was delivered via the air nozzle (Nylon, ~ 2.4 mm in inner diameter, covered with metal tube for protection against biting) next to the spout (Fig. 1c). The air was gated with a high-speed solenoid valve for gas (K2-100SF-02, Koganei Corporation, Tokyo, Japan). The chamber was 28 cm in width, 40 cm in depth, and 30 cm in height, excluding items on the box. The chamber wall was translucent white to scatter light from the reflector.

### Preconditioning for the learning task

As a preconditioning protocol, naïve animals were first trained to learn licking the spout to get drops of water in the operant conditioning chamber (Fig. 1c). To provide motivation for the preconditioning, the naïve animal was deprived of water for 19 h. Preconditioning started at 9 am and lasted for 1 h. During preconditioning, drops of water were delivered whenever the animal licked the spout. After preconditioning, the animal was returned back to the home cage and again deprived of water for 11 h. On the same day, habituation to the behavioural task started at 9 p.m. as described in the following section. In the following days, animals used for the experiments were basically deprived of water in the home cage.

### Behavioural task requiring self-timed actions

The behavioural task was designed to encourage animals to perform self-timed actions^7,8^ based on the presented cue in each trial (Fig. 1a). In a task trial, one of two randomly chosen sensory cues, called ‘short’ and ‘long’ cue (described below), was presented to indicate possible action time to obtain reward, which was called the R(+) time window (Fig. 1b). To suppress less-attentive actions, the animal was required to maintain the head inserted into the hole leading to the spout, without licking the spout to trigger a cue presentation (Fig. 1d,e). The required duration for the insertion was termed the holding time (T_holding_), and it was 2 s or randomly chosen from 1 to 3 s in each trial (Fig. 1e). The inter-trial-interval was decided by the guinea pig, and the possible minimum interval was the holding time. The time of first contact between animal and spout after the cue onset was recorded as the action time in the trial, with a 0.1 ms time resolution (Fig. 1e). If the action time fell in the R(+) time window, drops of water (~ 120 μL, depending on the body weight) were delivered from the spout immediately after the first contact (Fig. 1a). There was only one chance to obtain reward per trial. Animals were not required to discriminate between two cues to obtain reward in the initial day of the task, called habituation (Fig. 2a, Day 0). During the task, animals occasionally made no contact with the spout by the deadline of the trial, 3.5 s after cue onset (Fig. 1b), and it was counted as a abandoned trial. Other trials were referred to as performed trials. Each daily session of the task started around 9 pm and lasted for 12 h. The number of performed trials in a daily session was decided by the animal. The task was controlled with a TDT real-time processor (RP2.1 or RX6, Tucker-Davis Technologies, Alachua, FL, USA).

### Sensory cues

We used two auditory or two visual cues to indicate a R(+) time window for the cue (Fig. 1b). Differences in the sensory modalities of these cues had no significant impact on the dynamics of timing precision, once the animal had discriminated the cues. There were four variations of auditory cues, consisting of a narrow band noise with a frequency centred at 0.5, 1, 3.4, or 13.5 kHz. The bandwidth of these noises was 0.5 octaves (centre frequency ± 0.25 octave). The principle of synthesis of a calibrated band noise has been described in a previous study^30^. A visual cue consisted of diffused light from red LEDs or blue LEDs. Technical details are described in the Supplementary Methods.

### Animal health monitoring

The body weight of each guinea pig was measured every day before and after a daily experiment to monitor health conditions. If the task was too difficult for the animal, its body weight decreased due to the small amount of water intake during the task. A guinea pig’s water requirement is 10–14 mL/100 g body weight daily^31^. If body weight decreased > 10% compared to the weight in a previous day of experiment, a 1-day rest was given to the animal with enough water (~ 500 mL in the bottle) and food in the home cage.

### Quantification of timing precision

Although the Q factor has been widely used to quantify frequency precision or time precision of clocks^32^, the Q factor was not applicable to quantify timing precision in general timed actions because only a single action without periodic repetition could be sufficient for the action. Thus, we introduced a time version of Q factor, called Qʹ (cue-prime) factor, to describe timing precision in the population distribution of action time as defined in the following equation:

$$Q^{{\prime }} = \frac{T}{\Delta T}$$

where, T is timing achieved in the timed actions, or action timing (Fig. 7), and ΔT is error range of action time around T. The concept of the Qʹ factor is similar to the mean normalised by the standard deviation of the distribution in statistics. Although mathematical definitions of T and ΔT, e.g. mean and standard deviation of action time, can be as variable as the Q factor calculation^32^, Qʹ factors in different timed actions can be quantitatively compared if the same mathematical definitions of T and ΔT are applied in the comparison. To estimate the Qʹ factor at a specific moment from a temporal local distribution of observed action times without need for normality assumptions of the data distribution, we applied the median and interquartile range of the action times for the mathematical definitions. We first set a target trial for the performed trials to locally collect samples of action time (Fig. 3b, arrow). Next, we picked up the 19 samples of action time that were the closest in time for the same cue over 15 min backward from the target trial and defined a series of 20 consecutive samples of action time (Fig. 3b, filled circles). The median divided by the interquartile range (Fig. 3c) was the estimated Qʹ factor, called the local Qʹ factor. In practice, we determined 20 was a sufficient number because the local Qʹ factor qualitatively matched the trial-by-trial deviations of action time (Fig. 3a,e, arrows). If the number of performed trials to the same cue did not exceed 19 in the 15 min for the target trial, the local distributions of action time for the target trial were not analysed and the same analysis was attempted in the subsequent target trial.

### Data scrambling

Data representing action times in a daily session for the cue (**D**) comprised ‘time from experiment onset’ (ET) and ‘action time’ (AT) in each performed trial (Fig. 5a). If we consider a case in which the number of performed trials for the cue is represented by N and the data length is represented by N, then **D** in scrambled data (**D**_scrambled_) can be obtained from **D** in original data (**D**_original_) as described in the following equations:

$${\mathbf{D}}_{{{\text{original}}}} = \, \left\{ {\left\{ {{\text{ET}}_{{{1},}} {\text{AT}}_{{1}} } \right\}, \, \left\{ {{\text{ET}}_{{{2},}} {\text{AT}}_{{2}} } \right\}, \, \ldots , \, \left\{ {{\text{ET}}_{{\text{N}}} ,{\text{ AT}}_{{\text{N}}} } \right\}} \right\}$$

$${\mathbf{D}}_{{{\text{scrambled}}}} = \, \left\{ {\left\{ {{\text{ET}}_{{1}} ,{\text{ AT}}_{{{\text{RAND}}({1},{\text{N}})}} } \right\}, \, \left\{ {{\text{ET}}_{{2}} ,{\text{ AT}}_{{{\text{RAND}}({1},{\text{N}})}} } \right\}, \, \ldots , \, \left\{ {{\text{ET}}_{{\text{N}}} ,{\text{ AT}}_{{{\text{RAND}}({1},{\text{N}})}} } \right\}} \right\}$$

where ET_i_ and AT_i_ (i = 1 to N) are ET and AT of the i-th trial in **D**_original_, respectively. RAND(1, N) randomly assigns an integral number from 1 to N independently to each element of **D**_scrambled_ and AT_RAND(1,N)_ randomly refers to AT_i_ in **D**_original_. Because **D**_scrambled_ has the same set and order of ET with **D**_original_, the same number of local distributions can be extracted from **D**_original_ and from **D**_scrambled_.

## Supplementary information

Supplementary Figure Legends.

Supplementary Figure S1.

Supplementary Figure S2.

Supplementary Figure S3.

Supplementary Information.

## Data Availability

The datasets generated and/or analysed in the current study are available from the corresponding author on reasonable request.
