# Author Correction: Dynamic changes of timing precision in timed actions during a behavioural task in guinea pigs

**DOI:** 10.1038/s41598-021-94593-8

**Published:** 2021-07-27

**Authors:** Masataka Nishimura, Chi Wang, Reika Shu, Wen-Jie Song

**Affiliations:** 1 Department of Sensory and Cognitive Physiology, Faculty of Life Sciences, Kumamoto University, Kumamoto, Japan; 2 Program for Leading Graduate Schools HIGO Program, Kumamoto University, Kumamoto, Japan

Correction to: *Scientific Reports* 10.1038/s41598-020-76953-y, published online 18 November 2020

The original version of this Article contained errors.

In Figure 2c, the y-axis label “Abandoned ratio (%)” was incorrectly given as “Passed ratio (%)”.

Additionally, in the symbol legend, the unfilled circled indicating “abandoned ratio” was incorrectly given as “passed ratio”.

Furthermore, in Figure 4a, the x and y-axis labels were incorrectly swapped.

Lastly, in Figure 5a, the label indicating “Original” data was incorrectly given as “Real.”

In Figure 5f, the x-axis label “Original” was incorrectly given as “Real.”

The original Figures 2, 4 and 5 and their accompanying legends appear below.

**Figure 2:**
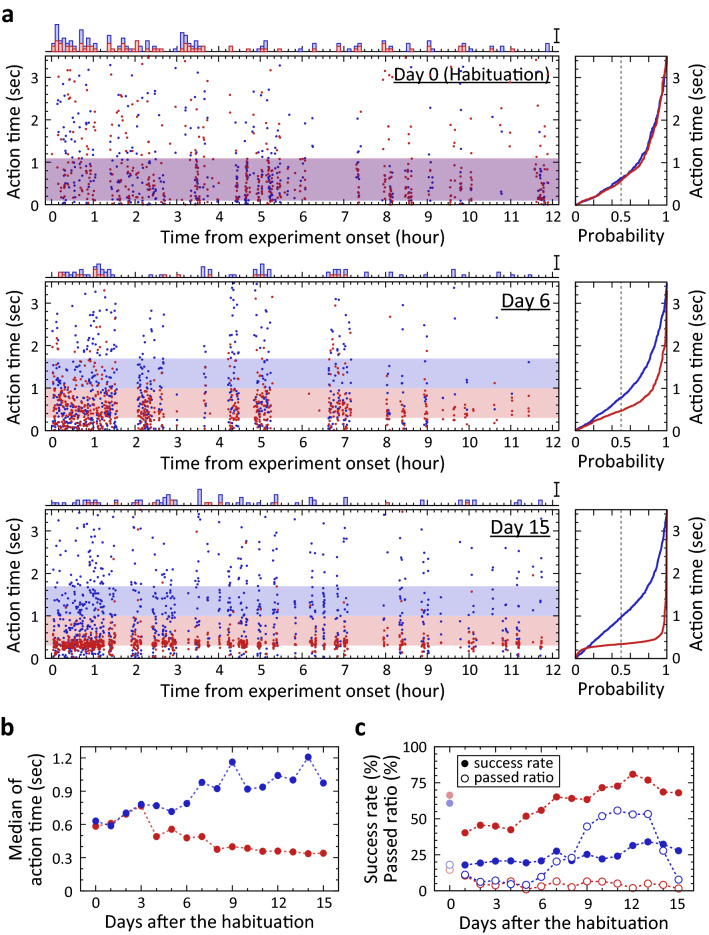
Learning process of the task in a representative guinea pig. (**a**) Action time for each trial of the task and its cumulative distribution. Left panels: Raster plots of action time on Days 0, 6, and 15. Red and blue dots indicate the time in ‘short’ and ‘long’ trials, respectively. This colour assignment for these cues is applied to all panels in this figure. The horizontal purplish band in Day 0 shows the overlapped R(+) time windows for ‘short’ cue and ‘long’ cue, 100–1100 ms. The horizontal reddish and bluish bands in Day 6 and Day 15 show R(+) time windows for ‘short’ cue and ‘long’ cue, respectively. These were 300–1000 ms and 1000–1700 ms, respectively. Histograms on each raster plot show the number of abandoned trials for each cue in the day. Bin width is 5 min. Scale bar is 5 abandoned trials. Right panels: Cumulative distribution of action time for each cue. The intersection of vertical dashed line and the probability curve is the median shown in (**b**). (**b**) Gradual separation of the two medians of action time for these cues in the guinea pig. (**c**) Change of success rate and abandoned ratio over days of experiment in the guinea pig. Filled and open circles indicate the success rate and abandoned ratio, respectively. The rate and ratio in Day 0 (habituation) are shown in faint colours.

**Figure 4:**
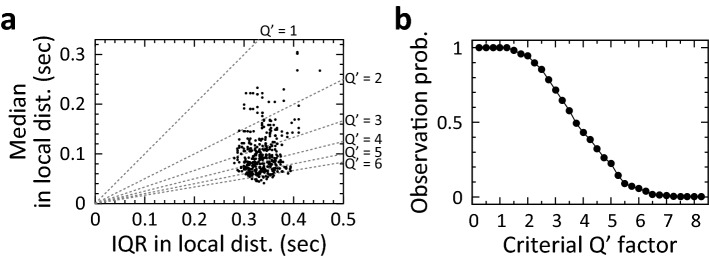
Calculation of the probability of observing a local distribution in which local Qʹ factor exceeds a local Qʹ factor threshold in a daily session. (**a**) Scatter plot of median and interquartile range (IQR) measured in each local distribution of action times in the same ‘short’ trials on Day 15 shown in Fig. 2a. The dashed line indicates median and IQR to be the local Qʹ factor. (**b**) Probabilities to observe a local distribution of which local Qʹ factor exceeds the local Qʹ factor threshold in the same daily session are shown in (**a**). The probability was calculated at 0.25 steps of local Qʹ factor threshold.

**Figure 5:**
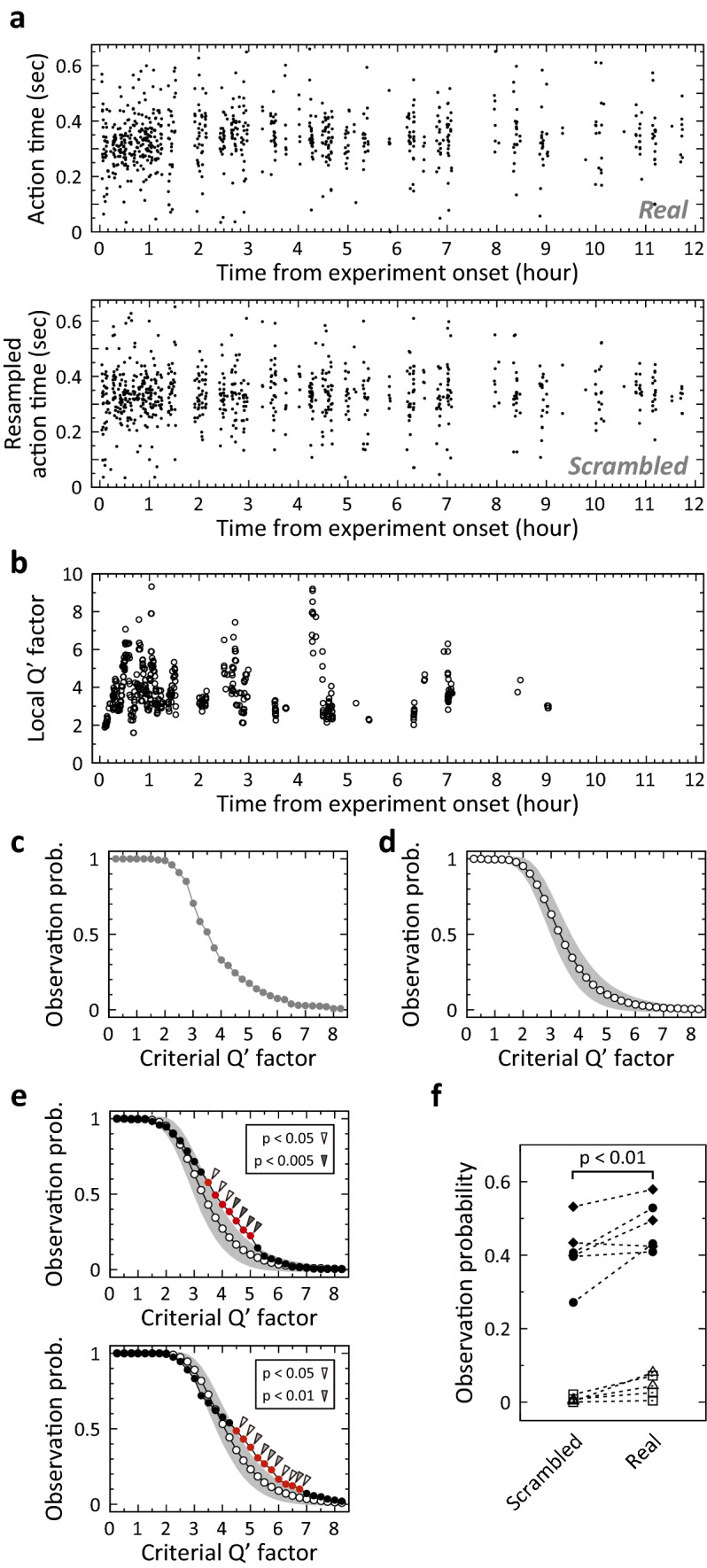
Statistical analysis of the observation probability by resampling the action time. (**a**) One resampling trial was obtained by data scrambling. The upper panel shows the observed action time in the original data. The lower panel is the resampled action time in scrambled data. (**b**) Extracted local Qʹ factors from the scrambled data shown in (**a**). (**c**) Probabilities to observe a local distribution of which local Qʹ factor exceeds the local Qʹ factor threshold in the scrambled data shown in (**a**). (**d**) Calculated possible range of observation probabilities under the assumption of no temporal change of timing precision. Open circle and greyish range indicate the mean of probability and the 95% confidence interval, respectively. The possible range was calculated from 10,000 sets of resampling and observation probability analysis. (**e**) Upper panel: A statistical analysis of the observation probability in a daily session obtained from a well-trained animal, compared to the possible range. The filled circle indicates the observation probability in original data that is shown in Fig. 4b. The possible range is shown in the same manner as (**d**). Significantly higher probability is marked by the arrowhead. Open arrowhead indicates p < 0.05. Double arrowhead indicates p < 0.005. Lower panel: The same examination in a different well-trained animal. The grey arrowhead indicates p < 0.01. (**f**) Comparison of probabilities to observe a local distribution of which local Qʹ factor exceeds 4 in scrambled data (left) and in original data (right), pooled from the four well-trained animals. One pair of probabilities linked with the dashed line was extracted from a daily session in an animal. Different symbols indicate different animals. The animals shown in (**e**) are represented by circle and diamond. The p-value was determined by the Wilcoxon signed-rank test (n = 11).

The original Article has been corrected.

