# Supplementary material for: Dynamic changes of timing precision in timed actions during a behavioural task in guinea pigs
Source: Sci Rep. 2020 Nov 18;10:20079. doi: 10.1038/s41598-020-76953-y (PMC7674413; doi:10.1038/s41598-020-76953-y)
Supplement: Supplementary file 1 — Supplementary Figure Legends. [file 41598_2020_76953_MOESM1_ESM.docx]

**Figure Legends**

**Fig. S1** Abandoned ratio and success rate in moving 2-hour analysis time-windows in a day of habituation.

**a** Abandoned ratios in moving 2-hour analysis time-windows. The onset and offset of analysis time-window was ±1 hour from time in the horizontal axis. The same data shown in Figure 2a (Day 0) was used for the analyses in (a) and (b). **b** Success rates in the moving analysis time-windows. **c** Left panel: Scatter plot of abandoned ratio and success rate shown in (a) and (b). Black dot indicates data from the early period in the day of habituation (1 - 3 hour as shown in (a) and (b) with arrows). Grey dot indicates other data in the day. r = -0.738 in data at the early period. Right panel: Another example of abandoned ratio and success rate in a day of habitation from another discriminated animal. r = -0.944 in data at the early period.

**Fig. S2** Temporal change of local Q’ factor in ‘short’ trials within a daily session in a well-trained animal.

Local Q’ factors in a daily session in a well-trained animal were plotted with the same manner in Figure 3h, obtained from a different animal. The onset and width of R(+) time window in ‘short’ trials were 200 msec.

**Fig. S3** Monte Carlo simulation showing variability of local Q’ factor without change in timing precision.

**a** Action time distribution of which probability density function is normal distribution with μ = 300 msec and σ = 60 msec, which was used for the simulation shown in (b). **b** Distribution of local Q’ factor sampled from the population shown in (a). To obtain the cumulative probability, sampling one local Q’ factor from the population was repeated 100,000 times in the simulation.
