# Supplementary material for: Dynamic changes of timing precision in timed actions during a behavioural task in guinea pigs
Source: Sci Rep. 2020 Nov 18;10:20079. doi: 10.1038/s41598-020-76953-y (PMC7674413; doi:10.1038/s41598-020-76953-y)
Supplement: Supplementary file 5 — Supplementary Information. [file 41598_2020_76953_MOESM5_ESM.docx]

Title:

**Dynamic changes of timing precision in timed actions during a behavioural task in guinea pigs**

Masataka Nishimura, Chi Wang, Reika Shu, Wen-Jie Song

**Supplementary Methods**

*Auditory cues*

We used narrow band noises for auditory cues to suppress the effect of resonance, which makes it difficult to deliver a pure tone with an expected sound intensity due to the nature of the standing wave, in the conditioning chamber. The sound signal was synthesised using a high-performance sound card for personal computers (SB-ZXR-R2, Creative Technology Ltd., Singapore) driven via the low-level audio interface (ASIO), or with a real-time processor (RX6 or RA16BA, Tucker-Davis Technologies, Alachua, FL, USA). A digital signal indicating onset of cue presentation was sent to the task controlling processor (RP2.1 or RX6, Tucker-Davis Technologies, Alachua, FL, USA) from the synthesiser. An analogue flip-flop circuit was used to digitize one channel of stereo output of the sound card and to hold the onset signal. A speaker (VS-SC8N, Visaton, Haan, Germany) attached to the conditioning chamber (Fig. 1c) was driven with a digital power amplifier (YDA138(D-3), Yamaha, Hamamatsu, Japan). To reduce the dark noise of the sound delivering system, an impedance-matched passive attenuator consisting of high wattage cement resistors (–14 dB) was inserted between the speaker and the amplifier. A frequency response of the sound delivering system was measured with a microphone (4191, B&K, Nærum, Denmark) placed at a 5-cm distance from the spout on middle line of the conditioning chamber at the level of entrance of guinea pig’s ear canal. Two of four narrow band noises were used as auditory cues in the task. The centre frequencies of these noises were 0.5, 1, 3.4, and 13.5 kHz. The bandwidth was 0.5 octaves (centre frequency ±0.25 octave). The sound intensity of one auditory cue was chosen from 60–72 dB SPL. Duration was 50 msec (10 msec cosine rise/fall and 30 msec plateau). When guinea pigs, especially naïve ones, showed non-specific impulsive responses to the cue [1], the sound intensity of the cue was decreased by 5–10 dB to suppress such non-specific responses.

*Visual cues*

We used red LEDs (NSPR510CS, Nichia Corporation, Anan, Japan) and blue LEDs (NSPB500AS, Nichia Corporation, Anan, Japan) for visual cues. Red and blue LEDs were alternately aligned in the 8 × 8 matrix of LED array. LEDs were driven by constant current circuits with a feedforward current control, which were designed and constructed in our laboratory, to improve reproducibility of light intensity waveform and to improve temporal response of light switching. To suppress potential variability of visual sensation, light from LEDs was first diffused with translucent rubber caps for LEDs and reflected on white paper on the roof (Fig. 1a, diffusing reflector). Then the diffused and reflected light was scattered by the translucent white wall of the conditioning chamber. Although the LED array has different lighting patterns in different visual cues, guinea pigs could not directly see the pattern from anywhere in the conditioning chamber (Fig. 1c, masking white paper). With this indirectly illuminating light, gaze control was not required for the presentation of visual cue. The light intensity of red LEDs and that of blue LEDs for visual cues were calibrated to be of the same physical intensity at the peak wavelengths (~625 nm and ~470 nm). A spectrometer was used to calibrate light intensity (C-700, SEKONIC, Tokyo, Japan). To suppress dark adaptation of guinea pigs, there was dim background light from the same LED array. The intensity of background light in each colour was 10 times weaker than that of light for visual cues. To equalize the absolute intensity of light in different conditioning chambers, we made a standard LED array with a fixed light intensity. Other LED arrays were then calibrated to the standard.

**Reference:**

[1] Suzuki TW, Kunimatsu J, Tanaka M. Correlation between Pupil Size and Subjective Passage of Time in Non-Human Primates. *J. Neurosci.* **36**, 11331-11337 (2016).
